# Global action plan for childhood diarrhoea: Developing research priorities: Report from a Workshop of the Programme for Global Paediatric Research

**DOI:** 10.7189/jogh.03.010406

**Published:** 2013-06

**Authors:** Alvin Zipursky, Kerri Wazny, Robert Black, William Keenan, Christopher Duggan, Karen Olness, Jonathan Simon, Evan Simpson, Philip Sherman, Mathuram Santosham, Zulfiqar A. Bhutta

**Affiliations:** 1 Program for Global Pediatric Research, The Hospital for Sick Children, Toronto, Ontario, Canada; 2 Department of International Health, Bloomberg School of Public Health, Johns Hopkins University, Baltimore, Maryland, USA; 3 Department of Pediatrics, St. Louis University, St. Louis, Missouri, USA; 4 Center for Nutrition, Division of GI/Nutrition, Boston Children's Hospital, Boston, Massachusetts, USA; 5 Department of Pediatrics, Case Western Reserve University, Cleveland, Ohio, USA; 6 Department of International Health, School of Public Health, Boston University, Boston, Massachusetts, USA; 7 PATH, Seattle Washington, USA; 8 Canadian Institutes of Health Research, Institute of Nutrition, Metabolism and Diabetes. Toronto Ontario Canada; 9 Division of Women and Child Health, Aga Khan University Hospital, Karachi, Pakistan.

## Abstract

**Background:**

Childhood diarrhoea remains a major public health problem responsible for the deaths of approximately 800 000 children annually, worldwide. The present study was undertaken to further define research priorities for the prevention and treatment of diarrhoea in low and middle income countries. We used the Child Health and Nutrition Research Initiative (CHNRI) process for defining research priorities. This provided a transparent, systematic method of obtaining the opinions of experts regarding research priorities in childhood diarrhoea. The present report describes the deliberations of a workshop that reviewed these research priorities by stakeholders including colleagues from: government agencies, academic institutions, major funding agencies and non–governmental organizations.

**Methods:**

The workshop included 38 participants, divided into four groups to consider issues in the categories of description, delivery, development and discovery. Each group received 20 to 23 questions/research priorities previously identified by the CHNRI process. Deliberations and conclusions of each group were summarized in separate reports that were further discussed in a plenary session including all workshop participants.

**Results:**

The reports of the working groups emphasized the following five key points: 1) A common theme was the need to substantially increase the use of oral rehydration salts (ORS) and zinc in the prevention and treatment of diarrhoea. There is a need for better definitions of those factors that supported and interfered with the use of these agents; 2) There is an urgent need to determine the long–term effects of chronic and recurrent bouts of diarrhoea on the physical and intellectual development of affected children; 3) Improvements in water, sanitation and hygiene facilities are critical steps required to reduce the incidence and severity of childhood diarrhoea; 4)Risk factors enhancing the susceptibility and clinical response to diarrhoea were explored; implementation research of modifiable factors is urgently required; 5) More research is required to better understand the causes and pathophysiology of various forms of enteropathy and to define the methods and techniques necessary for their accurate study.

**Conclusions:**

The participants in this workshop determined that use of the CHNRI process had successfully defined those research priorities necessary for the study of childhood diarrhoea. The deliberations of the workshop brought these research priorities to the attention of stakeholders responsible for the implementation of the recommendations. It was concluded that the deliberations of the workshop positively supplemented the research priorities developed by the CHNRI process.

Childhood diarrhoea is a major public health problem globally. Despite major advances in prevention and treatment more than 800 000 children still die every year of diarrhoea [1]. In addition to the high burden of mortality, the effects of diarrhoea in children who survive are many, including stunting, neurodevelopmental delay, concomitant infections, recurrent diarrhoea and failure to thrive as well as other social and emotional problems. In response to this persistent burden of global illness, we initiated a global action plan for childhood diarrhoea (“D–GAP”). As part of the D–GAP plan we developed an approach to define focused research priorities aimed at improving the study and the care of children with diarrhoea globally. The technique used to define research priorities was developed by the Child Health and Nutrition Initiative (CHNRI) and is referred to as the CHNRI method [2]. Our use of this method of assigning research priorities is described in detail elsewhere [3]. It includes recommendations of research priorities of over 150 experts in childhood diarrhoea from around the world. The CHNRI process permitted us to determine the importance of these questions and to assign a ranking to them. Previous studies of childhood diarrhoea [4,5] used the CHNRI process to define research priorities to reach the Millennium Development Goals by 2015. The current study determines research priorities for the next 15 years. As a supplement to the CHNRI process, a workshop was held to discuss the results of the CHNRI process and to consider further those steps necessary to reduce the ongoing burden of childhood diarrhoea in the world.

This paper describes the results of that workshop held under the auspices of the Programme for Global Paediatric Research (PGPR). The goal of the workshop was to review the research priorities determined by the CHNRI process by stakeholders including academics, clinicians, and representatives of major funding agencies and non–governmental agencies.

## METHODS

The PGPR workshop was undertaken to consider the results of the CHNRI process and to comment on the recommendations therein. The workshop was held on May1, 2012, in Boston, Massachusetts, USA, during the annual meeting of Pediatric Academic Societies. The one–day event included 38 participants all of whom were “stakeholders” since they are involved in the care and study of children with diarrhoea. Participants included colleagues from: government agencies (National Institute of Child Health and Development, Center for Disease Control, Fogarty International, Canadian Institute of Health Research, and USAID), World Health Organization, paediatric associations (International Pediatric Association, American Academy of Pediatrics), non–governmental agencies (PATH, Management Science for Health, UNICEF, International Rescue Committee, Micronutrient Initiative) and several academic institutions in, Bangladesh, Canada, Nigeria, Pakistan, United Kingdom and United States.

The program began with two reviews for orientation. The first lecture delivered by Dr Zulfiqar Bhutta who described the overall global action plan for diarrhoea. This was followed by a presentation by Dr Alvin Zipursky who reviewed the CHNRI process and the results arising.

The participants in the workshop formed four working groups, Discovery (New interventions), Description (Epidemiology), Development (Improving existing interventions) and Delivery (Health policy systems, including cost–effectiveness). These four groups were defined using the criteria previously described by Rudan et al [2].

Each group was given the top 20–23 questions (research recommendations) from their corresponding categories, identified by the CHNRI process (**Tables 1**** to ****4**). The groups were asked to review their list of research questions and consider issues raised by those questions. Following the deliberations of each group a report was prepared and presented in a plenary session of all workshop participants. Participants were invited to discuss the reports and issues raised. Following the workshop the group chairs were asked to prepare final reports which are considered in this article. The four groups were asked to consider the following when discussing each of their 20–23 recommendations:

**Table 1:** Discovery related questions identified by the CHNRI process

1	How do we improve the efficacy of live oral vaccines against gut or gut–acquired pathogens in low– and middle–income countries?
2	Develop successful vaccine against *Cryptosporidium*, *Shigella*, *Giardia*.
3	Research suggests that cognitive deficits associated with early childhood diarrhoea persist for at least 4 or 5 y. What measures can capture this deficit? How long does this deficit persist?
4	What are the fully burdened cost–benefits of different diagnostic technologies – molecular vs culture vs antigen detection vs microscopy?
5	What is the role of short–chain fatty acid delivery to the colon in enhancing sodium and water absorption, reducing fluid secretion and facilitating mucosal repair?
6	By what mechanisms (at gut and immunologic level) do malnutrition and various micronutrient deficiencies increase risk of severe diarrhoea?
7	Identify and validate biomarkers of “gut health” (eg, gut barrier function, inflammatory biomarkers, etc.) to identify those children at risk of chronic enteropathy.
8	What is the role of vitamin D deficiency in diarrhoea risk?
9	Study the effect of zinc on the gut secretory mechanisms.
10	What is the role of co–infections in childhood diarrhoea?
11	Can a water storage vehicle be developed with slow release halogen?
12	Can and should cheap and rapid diagnostic tests for common enteric pathogens be created for use in the field?
13	Will field or clinical use of rapid diagnostic tests for common enteric infections lead to improved accuracy of disease diagnosis (and more appropriately, targeted therapies or health measures)?
14	Will more rapid and accurate diagnosis of enteric diseases (and use of targeted therapies) improve measures of disease outcome and long–term health?
15	Will targeted therapies and new diagnostics decrease antibiotic resistance?
16	What is the effect of enteral glutamine on mucosal and systemic immune responses in children with diarrhoea?
17	Assess the utility of targeting NKCC, K channels and Na–coupled transporters in diarrhoea therapy.
18	What is the better approach to improve the intestinal microbiome in order to enrich the nutrient absorption and protect the intestinal barrier function following enteric infections?
19	Are there enteropathogens (particularly chronic infections for which treatment exists, ie, helminths) that modulate the incidence and severity of other enteropathogens?
20	Although mucosal immune responses are elicited by oral vaccines, responses to such vaccines may be relatively short duration compared to that induced by wild type disease. What are the reasons for this? What are the mediators and modifiers of long–term mucosal immunity? Would improved vaccines, regimens and/or immunization strategies result in longer duration? *V. cholerae* is a well studied, paradigmatic, non–invasive pathogen, and oral and live attenuated cholera vaccines, as well as subunit and conjugate vaccines exist. Could cholera be used as a mucosal model to address these questions? Such an approach may not only inform how to induce long–term immunity against mucosal pathogens as a group, but, if successful, could facilitate roll out and implementation of current or improved cholera vaccines.
21	Develop molecular techniques for understanding co–infections (bacterial and parasitic and viral causative agents).
22	How do age, aetiology and severity of diarrhoea affect the integrity of the gut and subsequent growth/health?
23	Establish the role of chloride channels in rotavirus–induced diarrhoea and then develop and test inhibitors of calcium activated chloride channels.

CHNRI – Child Health and Nutrition Research Initiative, KNCC – Na–K–Cl transporter

**Table 4:** Delivery–related questions identified by the CHNRI process

1	Identify and test alternative delivery strategies designed to ensure that ORS and zinc are reaching hard to reach populations and being used by the poorest of the poor (for example, home distribution of ORS and zinc).
2	What factors drive care–seeking behaviour during a childhood diarrhoea disease? How can we position ORS and zinc to best respond to these factors?
3	What factors most effectively drive caregiver demand for ORS and zinc?
4	What is the added impact of integrated community case management on early and equitable administration of appropriate treatment for acute diarrhoea?
5	Determine how the perception of diarrhoea as an illness affects: Key household practices like handwashing; Willingness to pay for point of use water disinfection products; Care seeking; and, Compliance to ORS and zinc treatment.
6	Determine how best to move caregivers from knowledge of ORS and/or zinc treatment to actual trial and eventual adoption as routine practice. Identify the stages of behaviour change in order to tailor messages accordingly.
7	Do we need to move from general and generic to more specific targeted messaging? When and what would this include?
8	To move a caregiver from awareness to trial of ORS and zinc, what will be the relative impact of mass media vs group vs one–on–one communication strategies? Does this vary by whether a rural or urban population?
9	What contextual or cultural factors positively or negatively influence ORS and zinc utilization or compliance?
10	Determine the best indicators for measuring the effectiveness of communication messages for childhood diarrhoea and the effectiveness of different communication channels in terms of a) awareness b) readiness to try, and c) actual use of ORS and/or zinc
11	Does the community–led total sanitation approach lead to decreased diarrhoea risk?
12	How best to effectively reduce the gap between knowledge and use of simple and effective interventions, such as ORS (eg, behavioural research, product improvements)?
13	What is the effect of promoting a strategy asking mothers to keep ORS packets and zinc at home for use in case of diarrhoea on use and coverage, when compared to the usual strategy that requires mothers to go to a CHW or a Health Facility to obtain ORS and zinc in case of diarrhoea?
14	Test indicators to determine effectiveness of IMCI and iCCM in reducing the burden of childhood diarrhoea.
15	Are ORT corners effective in reducing hospital admissions for severe to moderate dehydration?
16	What are the costs and benefits of the education measures to decrease diarrhoeal disease in the developing world?
17	Conduct social marketing research to improve acceptability of zinc treatment in the public and private sections – packaging, language, health messages.
18	Which strategies and messages are effective in convincing health care providers of the advantage of ORS and zinc compared to antibiotics or other drugs?
19	What factors, including mothers’ education, would influence acceptability of zinc supplementation and high/earlier use of ORS in the community?
20	What is the effectiveness of iCCM in increasing coverage of zinc and ORS?
21	Assess effectiveness of delivery strategies to provide zinc and ORS
22	Assessment of key knowledge gaps in community awareness of the relationship between nutrition and the occurrence of diarrhoea and the relationship between diarrhoea and long–term development in children.

CHNRI – Child Health and Nutrition Research Initiative, iCCM – Integrated Community Case Management, IMCI – Integrated Management of Childhood Illnesses, ORS – oral rehydration salts

What is the relative importance of each recommendation?

What steps are required for implementation of each recommendation?

Clarify the phrasing of each question/recommendation to improve implementation

Provide suggestions of groups or individuals who could implement specific recommendations.

How can global resources collaborate to implement specific recommendations?

What should be the long term goals of D–GAP to insure implementation of the recommendations?

Comment on the CHNRI process as a means of determining research priorities in the development of a global action plan for childhood diarrhoea.

## RESULTS

### The Discovery Group

This group analyzed the 23 questions listed in **Table 1** and recognized that they represented issues that could be presented to major funding agencies. The first area identified was an improved understanding of mucosal defence mechanisms. This is a fundamental research initiative concerning the regulation of innate and adaptive immune responses at the gut level and improved characterization of those factors that influence that response such as the microbiome and certain pathogens. A second major area worthy of additional research funding is the mechanism underlying enteropathy, including the effects of nutrition and the gut microbiome on intestinal function as well as repair of the bowel following either repeated bouts of acute diarrhoea or chronic enteropathy. The group also recognized the need for research in terms of the impact of enteric infections on long–term brain development, physical growth and metabolic consequences.

The group emphasized the need for improved diagnostics and technology to further the understanding of bowel function. This could include studies of intestinal motility as well as nutrient and fluid losses. In addition the Discovery Group identified a need for effective long–term studies of child health including weight and anthropometry as well as nutritional status and indices of child development. The development of biotechnology devices for specific markers of water quality, sanitation and food safety were also identified as research priorities.

### The Description Group

This group received 20 questions for appraisal (**Table 2**). They first considered recommendations regarding risk factors and elaborated on them for appraisal. For instance it was noted that sickle cell disease, which is very common in many low income countries could well be an important determinant of the severity of diarrhoea and resulting morbidity and mortality. Implementation of these issues was discussed ; it was felt that determining genetic risk factors such as sickle cell disease would be feasible either with a case–control or prospective study design. Nutritional risk factors could be examined through the new birth cohort studies. This group felt that data on the effect of HIV on diarrhoea are likely available now.

**Table 2:** Description related questions identified by the CHNRI process

1	What are the barriers against the appropriate use of ORT?
2	What factors have led to the decline in ORS use rates in countries where rates were high and now are low?
3	What are the attributes of successful and sustainable childhood diarrhoea programs? E.g. what have been the design and strategies used in programs and interventions where the burden of diarrhoeal diseases has been drastically reduced?
4	To what extent does the roll out of rotavirus vaccination reduce the burden of acute dehydration as well as all diarrhoeas?
5	What are the individual risk effects of malnutrition, poor sanitation, low level of education and reduced levels of vitamins and micronutrients in acquiring diarrhoea in children living in the developing world?
6	What are the developmental stages/ages at which children are most at risk of long–term cognitive impacts from diarrhoea? Is there a critical window for early childhood diarrhoea that can affect future physical and mental development (0–6 mo, 6 months – 2 years or 3–5 years)? (If it is greatest in the first six months to one year, one might place more emphasis on breast feeding and weaning practices)
7	Evaluate if early initiation and exclusive breastfeeding is associated with reduced burden of diarrhoea and improved growth.
8	Do access to, and benefits received, from nutritional supplementation programmes reduce global burden of diarrhoeal disease?
9	What are the risk factors for diarrhoea mortality?
10	What is the role of host factors in determining diarrhoea morbidity and mortality (eg, demographic, nutritional, genetic)?
11	What are the key transmission pathways and dominant pathogens of DD in different settings?
12	What is the sensitivity and specificity of the current home oral rehydration treatment and ORS questions in DHS and MICS and are there better questions to measure use of ORS?
13	What micronutrient deficiencies are risk factors for diarrhoea incidence or severity?
14	How does childhood diarrhoeal illness correlate with adult height? What is the impact of acute, prolonged, persistent and recurrent diarrhoea on growth trajectories of children in impoverished endemic areas?
15	What are the environmental and social/behavioural risk factors for diarrhoea?
16	What is the best current estimate of child mortality from diarrhoea globally and in various regions of the world?
17	Which pathogen is the most important cause of diarrhoea in target ages, seasons and regions?
18	What are the major bacterial, viral and parasitic pathogens responsible for mortality/morbidity in acute and chronic diarrhoea among children worldwide? Are there global monitoring systems?
19	How can we utilize data collected on childhood diarrhoea diseases to reduce rates of infection and disease? Can this data be used to help target the development of specific vaccines, or will vaccines actually be applicable? On the other hand, can these data be used to target areas for improved hygiene/sanitation to reduce incidence?
20	Develop and test and ordering algorithm for health worker/community workers/physicians for identifying causative agents of diarrhoea in an individual or outbreak situation (diagnostic test algorithm).

CHNRI – Child Health and Nutrition Research Initiative, ORS – oral rehydration salts, ORT – oral rehydration therapy

The importance of breast–feeding was recognized however it was felt that the focus for further studies should be on behavioural changes to increase breast–feeding rather than simply describing the effect of breast–feeding on diarrhoeal disease, for which there is ample evidence. Concerning recommendations on the aetiology of diarrhoea this group felt that current ongoing studies would likely provide sufficient information at this point.

This group then discussed the need for indicators to measure treatment practices and decided that this was a very high priority. The group also discussed various barriers and incentives for treatment provision. These included financial and household barriers, as well as equity issues including gender.

It was suggested that it would be important to determine the clinical characteristics of fatal cases in acute and persistent diarrhoea. Verbal autopsies were suggested as a means to understand the nature of fatal diarrhoea . This group also identified a need for studies of why diarrhoea mortality has declined worldwide. For example, data should be available in studies in Brazil where there has been a substantial drop in diarrhoea mortality [6]. Furthermore, it was suggested that there is a critical need for data on country specific diarrhoea mortality, incidence and severity.

### The Development Group

The Development Group divided the 21 assigned research questions (**Table 3**) into several categories and discussed the importance of the research questions. Under the category of food and nutrition, members of the group assigned high priority to the question of the effects of zinc supplementation on diarrhoea prevention as well as determining whether iron and other micronutrient supplements might impact the absorption or bioavailability of zinc. The group assigned a lower priority to determining which practices are most efficacious in improving the safety of food served to children 0 to 59 months at home.

**Table 3:** Development related questions identified by the CHNRI process

1	How do we improve the availability and uptake of interventions for diarrhoea that have consistently been shown to be effective (eg, the 2009 WHO 7–point plan)? Can a mixture of zinc and ORS be developed that successfully reduces duration and stool output?
2	Do interventions to support mothers (eg, reduce maternal depression, strengthen maternal coping, problem solving for child health) impact diarrhoeal disease outcomes? Provision of low cost/sustainable health education packages through community involvement (community motivation steps) to mothers to prevent diarrhoea and assess effects on children’s cognition and school achievement.
3	What is the impact of waterless hand sanitizer use on diarrhoea risk in household and school setting, particularly in water–constrained areas?
4	What are the critical times to wash hands to reduce diarrhoeal disease?
5	Could an ORS formula be developed that decreases output?
6	Evaluate calcium–supplemented ORS to reduce fluid secretion through enterocyte calcium receptors.
7	How might HWTS demonstration at ORT corners increase uptake and use of HWTS products and subsequent reduction of diarrhoeal disease incidence in mothers presenting with infant at ORT corners?
8	What is the best way to improve the microbial quality of the food served to children 0–72 months at home?
9	Assess the efficacy of zinc supplementation as adjunct to standard anti–*Shigella* treatment on the gut mucosal and systemic response.
10	What is the impact of intermittent water supply on DD and how can we ensure the microbiological quality of intermittent piped supply?
11	Develop age–appropriate, geography–appropriate, duration–appropriate (acute/chronic), and characteristic–appropriate (bloody/non–bloody) algorithms for management of different diarrhoea syndromes in different paediatric hosts.
12	What is the effect of intermittent therapy with zinc on diarrhoea prevention when given at routine contacts?
13	What are the triggers of handwashing behaviour change at different occasions and for different target groups (eg, parents, adolescents)?
14	What effect does the provision of sanitation and water supply in schools have on community behaviours with respect to sanitation and hygiene and what are the health outcomes for children in school and for the wider community?
15	What is the potential for women’s groups or peer–counselling/training of community–based cadres to improve infant/child nutrition and reduce diarrhoea through the update of preventive/therapeutic strategies?
16	Determine whether iron and other micronutrient supplements reduce the effectiveness of zinc to prevent diarrhoeal disease (RCTs).
17	In randomized controlled field trials in Sub–Saharan Africa and South and Southeast Asia, oral rotavirus vaccines have conferred ~ 50–60% efficacy. WHO SAGE has recommended their use and GAVI has committed to finance introduction of rotavirus vaccine into national EPIs. If a poor Sub–Saharan African country achieves a high coverage of rotavirus vaccine, is it conceivable that the indirect protective effects, in addition to the direct protection, may result in a greater than expected impact on diminishing disease burden? Should it be a high priority to affirm (or disprove) this hypothesis since it has important public health implications?
18	In view of clear reduced immunogenicity of oral enteric vaccines in children in developing countries, should significant resources be allocated to better understand the reason for such findings and for development of alternative modes of delivery (modified oral delivery and/or alternative routes) for efficient immunization with enteric vaccines in these populations?
19	There are two licensed non–living oral cholera vaccines that require two doses to immunize and are useful for control of endemic disease. For control of epidemic cholera, particularly in unsettled and emergency situations, should resources be applied to complete development and achieve licensure of one or more single–dose oral cholera vaccines?
20	Natural *Shigella* infection confers around 70–75% protection against the homologous serotype for a limited period of time ( ~ 2–3 years). This figure parallels the level of serum and antibody secreting cell (ASC) responses to natural infection. What would be a priority for investment of research resources: development of multicomponent (5–valent) vaccines, which will cover the most common serotypes reaching this extent of protective efficacy in developing countries? And/or (?) discovery of common protein antigens (perhaps secreted proteins in vivo) which will cross–react with *Shigella* homologous and heterologous sera and further study their immunogenicity and potential to cross protect?
21	Natural enterotoxinogenic *Escherichia coli* (ETEC) infection confers around 70–75% protection against the homologous strain for a limited period of time ( ~ 2–3 y). This figure parallels the level of serum and antibody secreting cell (ASC) responses to natural infection. What would be a priority for investment of research resources: development of multicomponent (eg, multivalent colonization factor antigen–based) vaccines, which will cover the most common antigenic types reaching this extent of protective efficacy in developing countries? And/or (?) the discovery of common protein antigens (perhaps secreted proteins expressed in vivo) which will cross–react with ETEC homologous and heterologous sera and further study their immunogenicity and potential to cross protect?

CHNRI – Child Health and Nutrition Research Initiative, EPI – Expanded Programme on Immunization, HWTS – household water treatment and storage, ORS – oral rehydration salts, ORT – oral rehydration therapy, RCT – randomised controlled trials, WHO SAGE – World Health Organization Strategic Advisory Group of Experts (SAGE) on Immunization

In the category of education, the highest priority was assigned to the question of whether the provision of low cost sustainable health education packages through community involvement to caretakers could serve to prevent diarrhoea and impact children's long–term cognition and school achievement. Assigned a lower priority was the question of whether trained groups within a community could improve infant and child nutrition and reduce diarrhoea through update of preventive and therapeutic strategies.

In the category of treatment this group assigned the highest priority to three questions:

1. Can an appropriate mixture of zinc – ORS be developed such that a sufficient dose is received by the child to reduce duration and stool output?

2. Could an ORS formula be developed that decreases stool output as well as improving hydration?

3. Does calcium–supplemented ORS reduce fluid loss? Of lower priority was the question of whether zinc supplementation could be used as an adjunct to standard treatment of acute bloody diarrhoea.

### The Delivery Group

This group divided the 22 questions (**Table 4**) into four major themes.

1. The use of behavior modification and communication strategies in the home and in the community

This group examined the drivers of prevention and barriers to treatment for reducing childhood enteric disease. The group determined that there is need for research to determine the most effective behavioural changes and communication strategies to increase utilization of treatment such as oral rehydration solution and zinc. Research questions in this group also addressed the behavioural changes in communication strategies necessary to promote important prevention strategies such as handwashing with soap, household water treatment, sanitation and nutrition. It was recognized that all of these included drivers and barriers such as poverty, physical inaccessibility, maternal education and cultural practices. This group also considered communication strategies such as household marketing, mass communication and social marketing.

2. The delivery of the products, including the role of health care workers, the available facilities and training

This group considered delivery of adequate health worker training as well as promotion of prevention and treatment strategies. Their recommendations consisted of various communication strategies, including marketing at the household level, mass communication and social marketing to promote health

3. Product improvements by both the private and public sectors

The Delivery Group found a need to evaluate innovative strategies to improve the acceptability palatability and positioning of ORS and zinc products. Members also determined that there is a need to monitor and evaluate new products, evaluate communication methods (packaging, language and health measures) and deliver these products effectively, through various pathways including private–public partnerships.

4. The cost of the products

Issues considered included an appraisal of how much are consumers in low and middle income countries willing to pay for zinc, ORS, water disinfection products, sanitation, rotavirus and measles vaccines?

Several questions, in addition to those that had been submitted, were added: What are the key knowledge gaps in community awareness of the relationship between nutrition and the occurrence of diarrhoea and the relationship between diarrhoea and long–term development in children? What are the appropriate tools to encourage appropriate use of antibiotics to treat the diarrhoea of childhood? What are the costs and benefits of the education measures, for both consumers and health care workers to decrease diarrhoeal disease in developing countries?

Also discussed were strategies to ensure that ORS and zinc as well as handwashing, household water treatment, nutrition, sanitation and vaccines are reaching hard–to–reach populations with emphasis on the poor. The group suggested that this would include the impact of Integrated Management of Childhood Illnesses (IMCI) for the appropriate treatment for acute diarrhoea. Other suggestions included oral rehydration therapy (ORT) corners, social marketing, commercial outlets, pharmacies, faith–based organizations, in addition to identifying the need to reduce cost barriers

## DISCUSSION

This report describes a workshop in which research priorities determined by the CHNRI process were then evaluated by a group of stakeholders. The CHNRI process provided a systematic, transparent method of obtaining and tabulating the opinions of experts regarding research priorities in childhood diarrhoea.

Kosek at al [5] note that the use of the CHNRI technique minimizes personal biases, however they state further: “there is a possibility that a different group, composed of many policymakers and program officers rather than the group that performed this exercise may yield somewhat different results”. Others have also commented on the need to include the opinion of stakeholders in an evaluation of the research priorities of the CHNRI process [7].

This workshop included participants involved in global child health who were able to consider proposed research priorities in relation to their own programs and experience. Furthermore the workshop provided an opportunity to consider the issues associated with implementation of the recommendations. Finally the workshop brought research priority issues to the attention of the participants, an important learning experience for those responsible for designing, implementing and funding programs aimed at reducing the global burden of childhood diarrhoea.

The deliberations and recommendations described in the Results section provide suggestions regarding priorities, related factors, additional recommendations and issues to be considered regarding implementation. It is evident that a major priority is the effective implementation of the use of ORS and zinc therapy. The Delivery Group discussed, in detail, the many issues that have to be considered to achieve optimal use of these agents including the importance of education as well as training both in the homes and in the community. Their recommendations constituted a veritable “plan for action” to ensure that this therapy reaches all those who require it.

It was recognized by several of the groups that the major concern internationally has been the high mortality of childhood diarrhoea. Noting the substantial reduction in diarrhoea mortality over the past decades, several groups emphasized the importance of also considering the long–term effects of diarrhoea in low and middle income countries, including malnutrition, physical and cognitive development as well as recurrent and chronic diarrhoea.

The Discovery Group recognized that many of the research priorities recommended by the CHNRI study relate to basic research and could be included in several major categories of research activities supported by national and international funding agencies. Furthermore this group described and emphasized the need for new technology to support the required basic and applied research.

The Description Group discussed the importance of risk factors significant in diarrhoeal mortality. For example, sickle cell disease, a common condition in Africa and India, can be a major factor in contributing to the lethal effects of diarrhoea. The group then recommended how this and other risk factors could be studied. This group also recognized the potential of learning more about the causes of death from diarrhoea by detailed studies of children who had died, including the use of verbal autopsies. It was also suggested that a study should be undertaken to determine the reasons for the recent reduction of diarrhoeal mortality worldwide.

Several groups discussed the importance of improving sanitation and water supply. In the plenary session there was agreement and emphasis of this as a most important topic, although it is a topic that has not had the general acceptance and priorities that it deserves.

All participants judged that the CHNRI process had successfully identified research priorities necessary for the study of childhood diarrhoea. The workshop contributed to the recommendations of research priorities provided by the CHNRI process in the following ways:

It clarified the phrasing of questions, a necessary step to further their eventual implementation.

It defined steps necessary for implementation of specific recommendations.

It brought the questions to the attention of stakeholders who are responsible for the implementation of such recommendations.

It provided an opportunity to discuss factors related to the individual questions: factors that have to be considered in the understanding of the recommendations for their eventual implementation.

Appreciation of the epidemiological issues related to an understanding of the global problem of childhood diarrhoea.

The inclusion of the recommendations of the workshop in the PGPR website permits ongoing discussion, recommendations and criticisms necessary for the continuing development of the research priorities necessary to solve the problem of childhood diarrhoea. The final complete reports of all workshop groups including a list of all participants can be found on the web site of the Programme for Global Paediatric Research (www.globalpaediatricresearch.com).

## CONCLUSION

The global action plan for diarrhoea required a means of determining research priorities for the future. The CHNRI process provided an objective and transparent method of providing these priorities. The workshop, described herein, provided input from stakeholders who are responsible for the implementation and evaluation of clinical and research programs aimed at reducing the global burden of childhood diarrhoea. Not only did the workshop serve to inform this group of research priorities but it also provided these individuals with further information on research priorities including avenues for funding and steps necessary for implementation.

The combination of the CHNRI process with input of stakeholders provides both a comprehensive education process and an in–depth discussion of the many issues involved in the implementation of research priorities to reduce the global problem of childhood diarrhoea.
